# Detection and quantification of zearalenone and its modified forms in enzymatically treated oat and wheat flour

**DOI:** 10.1007/s13197-023-05683-6

**Published:** 2023-02-15

**Authors:** Xenia Pascari, Stefan Weigel, Sonia Marin, Vicente Sanchis, Ronald Maul

**Affiliations:** 1 Applied Mycology Unit, Food Technology Department, University of Lleida, UTPV-XaRTA, Agrotecnio, Av. Rovira Roure 191, 25198 Lleida, Spain; 2 Department Safety in the Food Chain, BfR German Federal Institute for Risk Assessment, Max-Dohrn-Str. 8-10, 10589 Berlin, Germany; 3 Department of Safety and Quality of Milk and Fish Products, Max Rubner-Institut, Federal Research Institute of Nutrition and Food, Hermann-Weigmann-Straße 1, 24103 Kiel, Germany

**Keywords:** Zearalenol, MS/MS, QuEChERS, Sulfate conjugates, Amylolytic enzymes

## Abstract

An analytical method for the analysis of the mycotoxin zearalenone (ZEN) and its modified forms was developed. Sample preparation was performed based on a modified QuEChERS method combined with liquid chromatography coupled to a triple quadrupole mass spectrometry detection. The method was tested for linearity, precision, limits of detection and quantification and recoveries. The evaluation of the above-mentioned parameters was performed on oat flour. The method was applied to oat and wheat flours that were submitted to an amylolytic treatment (α-amylase and amyloglucosidase), similar to the one used in the cereal-based baby food production process. A decrease in β-zearalenol (β-ZEL) and β-ZEL-14-sulfate of approximately 40% after 90 min incubation was observed, the other analytes did not show any significant changes. To our knowledge, this is the first method that approaches the identification and assessment of ZEN-sulfate derivates in a cereal matrix.

**Supplementary Information:**

The online version contains supplementary material available at 10.1007/s13197-023-05683-6.

## Introduction

According to the survey performed by Mousavi Khaneghah et al. (2019), covering 24 years of investigation on mycotoxin occurrence, *Fusarium* mycotoxins were the most frequently encountered, deoxynivalenol (DON) and zearalenone (ZEN) being the predominant mycotoxins in cereals and cereal-based products.

ZEN is a non-steroidal oestrogenic mycotoxin, produced by several *Fusarium* species, mainly *F. graminearum*, *F. culmorum*, *F. crookwellense, F. cerealis, F. semitectum* and *F. equiseti,* which are common contaminants of cereal crops worldwide (Ismaiel and Papenbrock 2015). These fungi can contaminate cereals like maize, barley, wheat, oats, sorghum and, if the climate and subsequent storage conditions favour their growth and survival (increased humidity and temperature), they can produce high amounts of ZEN (Zinedine et al. 2007; Marroquín-Cardona et al. 2014). Due to its structural similarity with 17β-estradiol, ZEN was classified as a mycoestrogen compound (potent endocrine-disrupting chemicals which alter estrogenic signalling) (Kinkade et al. 2021). ZEN was proven as being able to induce various hormonal deregulations in humans and animals of both sexes, such as disruption of hormone management, strong embryonic toxicity, apoptosis, and oxidative stress in human embryonic stem cells etc. (Rogowska et al. 2019). Based on the exposure data, EFSA established a tolerable daily intake (TDI) of ZEN at 0.25 µg/kg body weight (EFSA 2011).

**Fig. 1:**
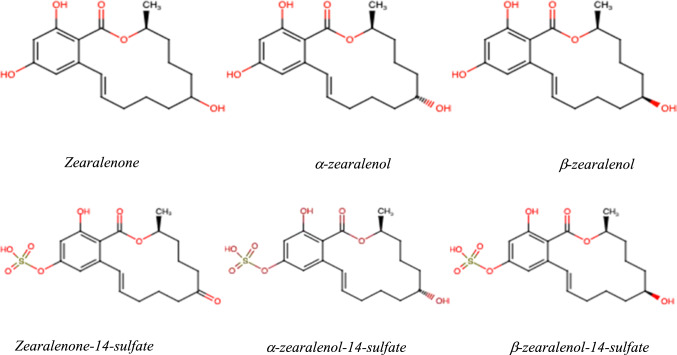
Chemical structure of zearalenone and its modified forms

In addition to the already known and thoroughly described fungal mycotoxins, modified ZEN mycotoxins have been detected in various cereals and cereal-based products, such as maize, oat, wheat, barley etc. (Bryła et al. 2018). The most commonly detected modified forms of ZEN are α- and β-zearalenol (α-ZEL and β-ZEL) and zearalenone-14-sulfate (ZEN-14-S), all found to be also natural fungal metabolites (Fig. 1) (De Boevre et al. 2013; Kovalsky et al. 2016). Also new modified forms of ZEN were identified e.g. by Brodehl et al. (2014), while studying the biotransformation of ZEN by the *fungi* of genera *Rhizopus* and *Aspergillus,* describing the formation of α-zearalenol-14-sulfate (α-ZEL-14-S). Unlike other modified *Fusarium* toxins such as acetylated or glycosylated DON, the molar ratio between free and modified forms of ZEN and ZEL is much more variable. Thus, no “safety factor” can be applied to calculate the total toxin burden from a measured content of the free form as it is suggested for DON.

Although, ZEN conjugates are proven to show a decreased estrogenicity (the intensity of the hormone-like response of ZEN that mimics the endogenous steroidal sex hormone 17β-estradiol activity after binding to estrogen receptors and effect the estrogen signalling pathways in animals), some modified forms are easily transformed into their parental form during mammalian digestion (Binder et al. 2017), thus contributing to the exposure to ZEN (Wang et al. 2019). The available studies suggest two possible ways of exposure to this toxin: (1) the consumption of contaminated cereals and cereal-based products or (2) the consumption of animal-derived products like meat or milk (Rogowska et al. 2019).

Until recently there were no available standards of ZEN sulfate-conjugates of sufficient purity, which made their quantification a challenge for the researchers. The available literature reports several high-performance liquid chromatography (HPLC) methods with (tandem) mass spectrometric (MS/MS) detection for the analysis of ZEN and its major modified forms (Baere et al. 2012; Brodehl et al. 2014). Considering European Food Safety Authority (EFSA) opinion on ZEN toxicity (EFSA 2011) that highlights the lack of sufficient information related to the modified forms and their toxic effects, the need in investigating the role of food processes involving naturally occurring and added enzymes become more evident.

Over the years, many sample preparation or mycotoxin extraction methods have been developed (Iqbal 2021). For instance, the immunoaffinity columns (IAC) and solid-phase extraction (SPE) cartridges are commonly used for their sensitivity and selectivity, nonetheless the range of mycotoxins that can be analysed using these sample preparation techniques is mostly limited to the parent mycotoxin (Berthiller et al. 2018; Sarkar et al. 2022). QuEChERS (Quick, Easy, Cheap, Effective, Rugged, Safe) extraction is frequently employed for the sample preparation in multi-mycotoxin analysis (Lu et al. 2013; Pereira et al. 2015). The main advantages of the technique are the fastness and good recoveries obtained for most of the mycotoxins. The present study aimed to develop a modified QuEChERS based method for sample preparation, followed by the simultaneous analysis of ZEN, α-ZEL, β-ZEL, ZEN-14-S, α-ZEL-14-S and β-zearalenol-14-sulfate (β-ZEL-14-S) using an HPLC system with mass spectrometric detection. The interest in the proposed method is valuable also due to its good performance in the case of ZEN- and ZEL-sulfates for which internal standards (IS) are not yet available. To our knowledge, this is the first study integrating the simultaneous identification and the quantification of three sulfate-conjugates of ZEN. The analytical method was validated in the oat flour matrix. Such a method is required to test the effect of the enzymatic (amylolytic) treatment of the oat and wheat flour during cereal-based baby food production process on the concentration of ZEN and its modified forms. Cereal-based baby food is a highly important part of the human diet during the first years of life (European Commission 2006a). Its production process aims to partially breakdown the starch contained in the cereals and facilitate the assimilation of the nutrients by the digestive system of a child. Amylolytic enzymes are the ones usually used to perform this task, mainly due to the high efficiency of the reaction (Martinez and Gómez 2016). Oats are often included in the list of ingredients in the cereal-based baby food production process, mostly due to their nutritional advantage compared to other cereals, such as the presence of functional proteins, $\beta$-glucans, lipid, starch, and a varied range of phenolic compounds with antioxidant activity (Rasane et al. 2015). The maximum level of ZEN in cereal-based baby food and cereals intended for its production were set at 20 µg/kg in the European Union (European Commission 2006b). Considering the possibility of sulfate-conjugates formation in cereals such as oat and wheat (Bryła et al. 2018) and the scarce knowledge related to the toxicity of modified and masked forms of ZEN (Liu and Applegate 2020), the development of a simple and accurate analytical method which allows the quantification of these compounds is of particular importance. Furthermore, the behaviour of conjugated forms of ZEN and ZEL during infant food processing should be investigated to unveil a potential rise of estrogenicity by conjugate cleavage during enzymatic food processing.

## Materials and methods

### Chemicals and reagents

ZEN, α-ZEL, β-ZEL and their U-[^13^C]-labelled standards were purchased from Romer Lab Diagnostics (Tulln, Austria). ZEN-14-S ammonium salt, α-ZEL-14-S sodium salt and β-ZEL-14-S sodium salt were acquired from ASCA GmbH (Berlin-Adlershof, Germany). Water was obtained from a Milli-Q® SP Reagent water system from Millipore Corp. (Brussels, Belgium). Methanol and acetonitrile as well as magnesium sulfate, formic acid and ammonium formate were purchased from Merck (Darmstadt, Germany).

### Samples

The oat and wheat flours were purchased in a supermarket in Lleida (Spain). The *Fusarium graminearum* strain (F.46) used for the present study was obtained from the strains collection of the Food Technology Department of the University of Lleida (Spain).

Before the inoculation, the flours were placed into ISO bottles (borosilicate glass 3.3 graduated bottle with ISO thread) and autoclaved. Then, they were aseptically transferred into Petri dishes (20 g of flour each) and 2 mL of sterile Milli Q water was added to each of them to reach a water activity suitable for the fungal growth (approximately 0.98–0.99). The prepared Petri dishes were stored at 4 °C overnight, water activity being measured with Aqualab Serie 3 TE (Decagon Devices, Washington, USA). One millilitre of *F. graminearum* spore suspension (10^6^ spores/mL) was added to the flour and slightly mixed for a better spreading of the contamination within the Petri dish. The portions were stored at 25 °C for 21 days. The obtained contaminated flour was dried at 40 °C, homogenized and the resulting mycotoxin concentration was determined.

The samples were submitted to treatment with two amylolytic enzymes (α-amylase and glucoamylase), under the conditions applied during cereal-based baby food production process. Briefly, 30 g samples were prepared by mixing a respective amount of the contaminated flour with the roasted blank flour. The mix was placed in a 100 mL beaker and the enzymes were added. Each of the enzymes was added in two dosages to the samples: 2.1 and 4 g of enzyme/kg of cereal flour. The 53 °C temperature was chosen for the present study as it corresponds to the temperature allowing for the activity of both enzymes. After the flour and enzyme mix was ready, 70 mL of distilled water at 53 °C was added and the slurry was put into a water bath for 10, 50, and 90 min. Samples for each treatment were prepared in parallel and per triplicate for every designed setup. When the time expired, the samples were rapidly cooled in an ice bath, frozen and lyophilized (Telstar LyoBeta 15, Terrassa, Spain). The obtained lyophilized samples were then packed individually and stored in a dry place at room temperature until analysis.

### Instrumental conditions

Detection and quantification of the analytes was achieved using a Shimadzu Nexera X2 HPLC system equipped with a binary pump and a thermostatic autosampler (Kyoto, Japan), coupled with a triple quadrupole mass spectrometer Sciex QTRAP 6500 + (Sciex Germany GmbH, Darmstadt,). Data acquisition and processing was performed using Analyst® and MultiQuant® software (Sciex Germany GmbH, Darmstadt, Germany). Separation was achieved at 40 °C on Restek Raptor Fluoro phenyl 100 × 2.1 mm, 5 µm column (Bellefonte, Pennsylvania, USA).

The mobile phase consisted of water (A) and methanol (B), both containing 0.1% formic acid and 300 mg/L ammonium formate, which was supplied at a gradient with a flow rate of 0.5 mL/min. The gradient was set as follows: 2% B for 0.8 min, after 4 min B was increased to 53%, after 6 min B was set at 60%, after 11 min B was 95% for 1 min, followed by 5 min of restoration to the initial conditions. Total run time was 17 min.

The detector was operated in negative electrospray ionization (ESI) mode under multiple reaction monitoring (MRM). Operating ESI conditions were setup as follows: curtain gas: 40 psi, gas 1: 60 psi, gas 2: 35 psi, collision gas flow: medium and source temperature: 300 °C. Ion spray voltage (IS) was set at 4500 V and − 4000 V in positive and negative ionization mode, respectively. Two characteristic ions were chosen for the assessment of the mycotoxins in the samples, quantification and the qualitative confirmation of the analytes. Table 1 regroups the MS/MS parameters applied. Figure 1 of the Supplementary Materials section showcases a chromatogram containing the six analytes.

**Table 1:** MS/MS parameters for zearalenone (ZEN), α-zearalenone (α-ZEL), β-zearalenone (β-ZEL), α-zearalenone-14-sulfate (α-ZEL-14-Sulf) and β-zearalenone-14-sulfate (β-ZEL-14-Sulf)

Mycotoxin	Retention time, min	ESI^1^ mode	Precursor ion (m/z)	Product ions (m/z)	Collision energy (eV)	Declustering potential (V)
ZEN	6.0	ESI-	317.1	131.0	− 40	− 75
				175.0	− 34	− 75
α-ZEL	5.9	ESI-	319.1	174.0	− 37	− 80
				160.0	− 44	− 80
β-ZEL	5.3	ESI-	319.2	174.0	− 37	− 80
				160.0	− 44	− 80
ZEN-14-S	4.8	ESI-	397.1	317.1	− 25	− 30
				175.0	− 46	− 30
α-ZEL-14-S	4.8	ESI-	399.1	319.1	− 25	− 70
				275.0	− 42	− 70
β-ZEL-14-S	4.3	ESI-	399.2	319.1	− 30	− 70
				275.1	− 44	− 70

The optimization of the compound related signal was performed by a direct infusion of the standards of ZEN-14-S and α-ZEL-14-S at a concentration of 10 µg/L (dissolved in methanol:water, 1:3, *v/v*) into the MS/MS detector.

### Procedure

#### Calibration solutions

To improve the accuracy of the measurements, for the analytes for which U-[^13^C]-labelled homologues were available, standard isotope dilution assay (SIDA) was performed. In the case of the sulfates derivates, matrix-matched calibration was carried out. For that, several blank oat samples were extracted by weighting 5 g of oat flour and mixing it with 10 mL acetonitrile and 10 mL water, shaking for 30 min (Multi Reax, Heidolph Instruments GmbH and Co.KG, Schwabach), centrifuging at 3800 g for 30 min (Megafuge 16, Thermo Fisher Scientific, Braunschweig, Germany), collecting 1 mL of the supernatant and mixing it with 250 mg of anhydrous magnesium sulfate. Then, the samples were centrifuged at 17,000 g for 5 min (Microfuge R, Beckmann, Krefeld, Germany) and all the resulting extract collected in a vial for further use.

Finally, three eight-point series of calibration solutions were prepared: one containing ZEN (8.008–500.5 ng/mL), α-ZEL (1.1047–69.074 ng/mL), β-ZEL (2.776–173.5 ng/L) and their respective U-[^13^C]-labelled homologues, dissolved in acetonitrile:water (25:75, *v/v*); one containing a series of calibration solutions of ZEN-14-S (0.858–546.25 ng/mL) and another one containing α-ZEL-14-S (0.853–947.1 ng/mL) and β-ZEL-14-S (0.6092–676.35 ng/mL), both dissolved in blank matrix extract (25%).

#### Sample preparation

QuEChERS method based on the European Standard EN 17279:2019 (Slovenian Institute of Standardization, 2019), describing an LC–MS/MS method for the screening of mycotoxins, was used for the preparation of the flour samples for analysis in the HPLC. Briefly, 5 g of sample were weighted in a 50 mL polypropylene tube, mixed with 20 mL acetonitrile/water (50:50, *v/v*), shaken for 30 min and centrifuged for 30 min at 3800 × g. Then, 1 mL of the supernatant was taken and mixed with 100 µL of the internal standard solution and 100 µL of Milli Q water. Afterwards, 250 mg of anhydrous magnesium sulfate was added, vortexed for another 30 s, centrifuged for 5 min at 17,000 × g and 200 µL of supernatant were diluted with 400 µL water and submitted to HPLC–MS/MS analysis.

#### Validation

For the validation of the method, blank oat flour samples (2.5 g) were spiked with ZEN and its modified forms and left overnight at ambient temperature to allow the evaporation of the solvent and the establishment of an equilibrium between the analytes and the matrix. The sample preparation procedure (“Sample preparation”) section was carried out on one concentration level and repeated three consecutive days with 3, 3 and 10 replicas on each day, respectively. The spiking levels of the blank oat flour was 2.5 µg/kg, 3.5 µg/kg and 3.7 µg/kg for β-ZEL-14-S, α-ZEL-14-S, and ZEN-14-S, respectively.

#### Data evaluation

Limits of detection (LOD) and limits of quantification (LOQ) were calculated according to (Wenzl et al. 2016), using the following equations:

$$LOD = 3.9*\frac{{s_{y,b} }}{b},\quad LOQ = 3.3*LOD$$

where s_y,b_ – standard deviation of the (pseudo)blanks’ concentration used for the determination of LOD and LOQ; b – slope of the calibration curve.

Also, intra- and inter- day precision were calculated. The significance in the changes of toxin concentrations was assessed for each flour and each enzyme dose, performing ANOVA and a post hoc Tukey HSD tests using Python 3.9.7 (α = *0.05*).

## Results and discussion

### MS/MS detection

MS/MS parameters (selection of the most abundant MRM transition, declustering potentials, collision energies) for ZEN-14-S, α- and β-ZEL-14-S were optimized using the automatic routine provided by Analyst in the negative ESI mode. The parameters for the other analytes have been optimized previously. Table 1 summarizes the set conditions of the optimized MRM transitions. ZEN-14-S and α-ZEL-14-S showed a relatively poor chromatographic separation on a regular C_18_-HPLC-column, which together with the similarity of the ionization and an insufficient specificity of the most abundant product ions between ZEN-14-S and α-ZEL-14-S, required two different spiked series of oat flour to be prepared: one containing ZEN-14-S and another one containing ZEN, α-ZEL, β-ZEL, α-ZEL-14-S and β-ZEL-14-S. The product ions selected for the identification and quantification of α- and β-ZEL-14-S (Q: 399.1 → 319.1 and q: 399.1 → 275.1) correspond to the ones described by Brodehl et al. (2014). However, they differ only by two amu from two of the most abundant transitions observed for ZEN-S ([M-H]^−^: 397.1 amu). Thus, ZEN-S containing one or two naturally occurring heavier isotopes interferes with the α-ZEL-14-S quantification. According to the natural isotope distribution mainly driven by the most frequently occurring ^13^C carbon and ^34^S atoms, the relative frequency of a ZEN-S molecule being two amu heavier than the most abundant molecule containing only ^12^C and ^32^S is approximately 8.1%. Since the two interfering compounds together were present in the samples that were to be analysed, for the quantification of ZEN-14-S and α-ZEL-14-S a different approach was applied. The ionisation and fragmentation behaviour of ZEN-14-S and α-ZEL-14-S is different and the most abundant heavier isotopic form ^34^S is cleaved and no longer part of the detected main MS/MS fragment ion. Thus, the relative natural abundance of the + 2 amu ZEN-14-S signal does not correspond to 8.1% to the signal abundance of α-ZEL-14-S. The abundance needs to be elucidated empirically. As a result of the optimization procedure, it was verified that the ionization of the ZEN-14-S leads to the constant formation of the product ions also characteristic for α-ZEL-14-S. Thus, since the two compounds were present together in the samples to be analyzed without being chromatographically resolved, a mathematical subtraction of the part of the area of the signal produced by ZEN-14-S from the area of the α-ZEL-14-S was required. Consequently, individual solutions of the two sulfate derivates of ZEN, at three different concentration levels (2, 5 and 10 µg/L in the spiked blank sample extract and liquid standard solution with 25% acetonitrile) were injected into the system. The four product ions were monitored on the ZEN-14-S chromatograms and the ratio of the areas of the quantifier ion for α-ZEL-14-S/area of the quantifier for ZEN-14-S was calculated to be around 2.9% at all concentrations (Supplementary materials, Table 1). The following formula was used to determine the true final area of the α-ZEL-14-S quantifier ion after subtraction of the ZEN-14-S induced interference:

$$Final\, A_{{ - {\text{ZEL}} - 14 - S}} = A_{{ - {\text{ZEL}} - 14 - S}} - \left( {2.9\% *A_{ZEN - 14 - S} } \right),$$

where *Final A*_α-*ZEL*-14-*S*_ – the resulting corrected area of α-ZEL-14-S peak; *A*_α-*ZEL*-14-*S*_ –the area of α-ZEL-14-S peak recorded by the MS/MS detector and *A*_ZEN-14-*S*_ – peak area of ZEN-14-S. The concentration of α-ZEL-14-S was determined by substituting the obtained final area in the linear regression equation.

### Method performance

The QuEChERS based method used for the present study showed relatively good results (Table 2). To account for possible interferences from the oat flour matrix, stable isotope dilution assay was used for the assessment of the concentration of ZEN, α- and β-ZEL. For the determination of ZEN sulfate-derivates (ZEN-14-S, α- and β-ZEL-14-S), for which the use of internal standard was impossible, matrix-matched calibration plots were constructed. The linearity was interpreted graphically using a scatter plot and confirmed through Mandel test for all analytes. The calibration curves for each analyte were also characterized by the coefficient of determination (R^2^) with values above 0.98. The precision was evaluated in terms of relative standard deviation (RSD). It ranged between 2.5 and 6.4% for intra-day precision (RSDr) and from 2.5 to 10.3% between the 3 days (RSDR). Method recoveries were in the range between 72.4 and 85.7% for ZEN sulfate-derivates and from 90.7 to 95.6% for other analytes studied. The LOQ for the different analytes ranged between 1.0 for β-ZEL-14-S and 59.1 µg/kg for ZEN.

**Table 2:** Overview of the limits of detection (LOD), limits of quantification (LOQ), recovery (%), intra- and inter-day precision (RSD_r_ and RSD_R_, respectively) and the coefficient of determination (R^2^) of the analytical method validated in oat flour (*n* = 16)

Mycotoxin	LOD (µg/kg)	LOQ (µg/kg)	Recovery,%	RSD_r_, %	RSD_R_, %	R^2^
ZEN	17.9	59.1	95.6	2.5	2.6	0.9995
α-ZEL	5.6	18.46	92.6	2.9	3.7	0.9979
β-ZEL	12.16	40.14	90.7	2.5	2.5	0.9988
ZEN-14-S	0.72	2.39	72.6	6.4	10.3	0.9984
α-ZEL-14-S	0.31	1.03	72.4	2.9	3.5	0.9831
β-ZEL-14-S	0.3	1.0	85.7	3.5	7.0	0.9813

### Samples analysis

Various studies have reported the presence of mycotoxins in cereal-based baby food (Dombrink-Kurtzman et al. 2010; Cano-Sancho et al. 2011; Juan et al. 2014).

**Fig. 2:**
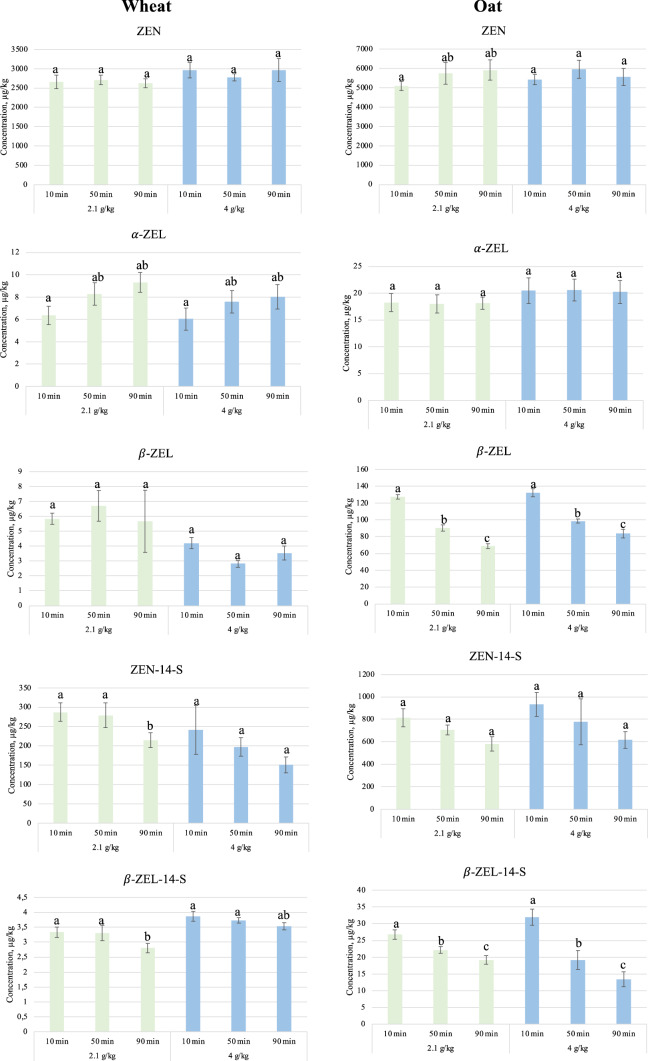
Changes in concentration (µg/kg ± SD) of zearalenone (ZEN) and its modified forms α-zearalenol (α-ZEL), β-zearalenol (β-ZEL), zearalenone-14-sulfate (ZEN-14-S) and β-zearalenol-14-sulfate (β-ZEL-14-S) in wheat and oat flour during enzymatic treatment at a dose of 2.1 and 4.0 g of enzyme/kg of flour during 10, 50 and 90 min. Tukey HSD test with compact letter display was run for each enzyme concentration separately. The different letters represent a significant change in mycotoxin concentrations between the treatments i.e., the farer the letters the bigger the numerical difference between the values (e.g. a is significantly different from b, but not different from ab etc.)

The developed method was applied to *Fusarium* contaminated wheat and oat flours, which were submitted to enzymatic treatment with α-amylase and amyloglucosidase (enzymes characteristic for cereal-based infant food production process). The aim was to investigate the effect of different incubation times on ZEN and its modified forms. Since infants are particularly sensitive to the effect of cereal contaminants and especially the ones targeting their in-development hormonal system, a special attention was given to following the changes in the levels of α-ZEL and α-ZEL-14-S. Figure 2 is a graphical representation of the obtained results. As the oat and wheat flours used in this work were infected in the laboratory, all the metabolites studied were also produced by the *Fusarium graminearum* strain used. The starting levels of ZEN, before the enzymatic treatment were 2660 µg/kg in wheat and 5095 µg/kg in oat flours, which is approximately between 10 and 878-fold higher compared to the levels of its modified forms in wheat, and between 6 and 295-fold in oat flour.

Regarding the enzymatic treatment of the samples, ZEN and α-ZEL are not showing any significant change during the applied process in none of the two matrices, although a slightly increasing trend can be observed. For β-ZEL, a significant decrease can be observed during the treatment at both concentrations of enzymes, 2.1 and 4.0 g enzyme/kg flour in oats with up to 46 and 37% after 90 min of incubation, respectively. In wheat flour, β-ZEL level did not show a significant change during the treatment.

Regarding the fate of ZEN sulfato-derivates, ZEN-14-S is found to be the most abundant in both wheat and oat. ZEN-14-S, starting at concentrations of 241.2 µg/kg in wheat and 815.6 µg/kg in oat, shows a decrease after 90 min at both doses of enzymes (up to 37% in oat and up to 29% in wheat flours), nonetheless it could not be proven statistically significant. The variable results for ZEN do not allow for claiming a correlation between the opposite behaviour of the two toxins. α-ZEL-14-S was not identified in any of the analysed samples. β-ZEL-14-S showed a very similar behaviour compared to its free form (β-ZEL) in both matrices, namely neither an increase nor a reduction compared to the initially identified levels of the toxin were recorded in wheat and a slight decrease in oat.

There is scarce literature on ZEN modifications during food production processes. Only a recent study published by Ksieniewicz-Woźniak et al. (2021) identified a *F. culmorum* strain that was able to biosynthesise ZEN-14-S with higher yield as compared to ZEN in wheat. In the study, afterwards the cereal was used submitted to a malting process. At the end of the malting the levels of both ZEN-14-S and ZEN increased significantly, which was explained by the natural contamination of wheat cultivars and the conditions during malting process appropriate for mycotoxin production. However, no conclusion on a potential interconversion of ZEN-14-S and ZEN could be drawn. Together with the data obtained in the present assay, the study performed by Ksieniewicz-Woźniak et al. (2021) show the need for a broader investigation of the *Fusarium* potential to produce ZEN and its sulfated metabolites.

## Conclusions

The developed LC-MS/MS method for the analysis of ZEN and modified forms demonstrated good performance criteria and applicability. The QuEChERS based method used for sample preparation also proved itself suitable for the quantification of the analytes showing good recoveries. The very similar ionization products of ZEN-14-S and α-ZEL-14-S and their poor separation on a regular stationary phase could requires an indirect calculation of the concentration which is feasible also in matrix samples as shown above, as long as a chromatographic separation is not achieved. The analysis of wheat and oat flours after the enzymatic treatment did not show any changes for ZEN and α-ZEL levels, however up to 46% decrease of β-ZEL was observed after 90 min of incubation. A quite similar behaviour was observed for ZEN-14-S and β-ZEL-14-S, which proves no correlation between the change of the two modified forms under the performed treatment. α-ZEL-14-S was not identified in any of the analysed samples.

In conclusion, the observed changes in the targeted analytes during the amylolytic treatment of the flours did not show a significant reduction nor elimination from the process. There are few studies on exposure to ZEN in infants, thus, monitoring the modified forms, of which the toxicity and the metabolism in the human body are not fully understood, is of great importance and especially the presence of the highly estrogenic α-ZEL derivatives require more attention from the researchers.

## Supplementary Information

Below is the link to the electronic supplementary material.

Supplementary file1 (DOCX 398 KB)

Supplementary file2 (DOCX 16 KB)

## Data Availability

The dataset used and/or analysed in this study are available from the corresponding author on reasonable request.

## List of symbols

ZEN: Zearalenone
α-ZEL: α-Zearalenol
β-ZEL: β-Zearalenol
ZEN-14-S: Zearalenone-sulfate
α-ZEL-14-S: α-Zearalenol-sulfate
β-ZEL-14-S: β-Zearalenol-sulfate
DON: Deoxynivalenol
QuEChERS: Quick, Easy, Cheap, Effective, Rugged and Safe
ESI: Electrospray ionisation
MRM: Multiple reaction monitoring
SIDA: Stable isotope dilution assay
LOQ: Limit of quantification
LOD: Limit of detection
RSD: Relative standard deviation
