# Supplementary material for: Detection and quantification of zearalenone and its modified forms in enzymatically treated oat and wheat flour
Source: J Food Sci Technol. 2023 Feb 15;60(4):1367–75. doi: 10.1007/s13197-023-05683-6 (PMC10020390; doi:10.1007/s13197-023-05683-6)
Supplement: Supplementary file 1 — Supplementary file1 (DOCX 398 KB) [file 13197_2023_5683_MOESM1_ESM.docx]

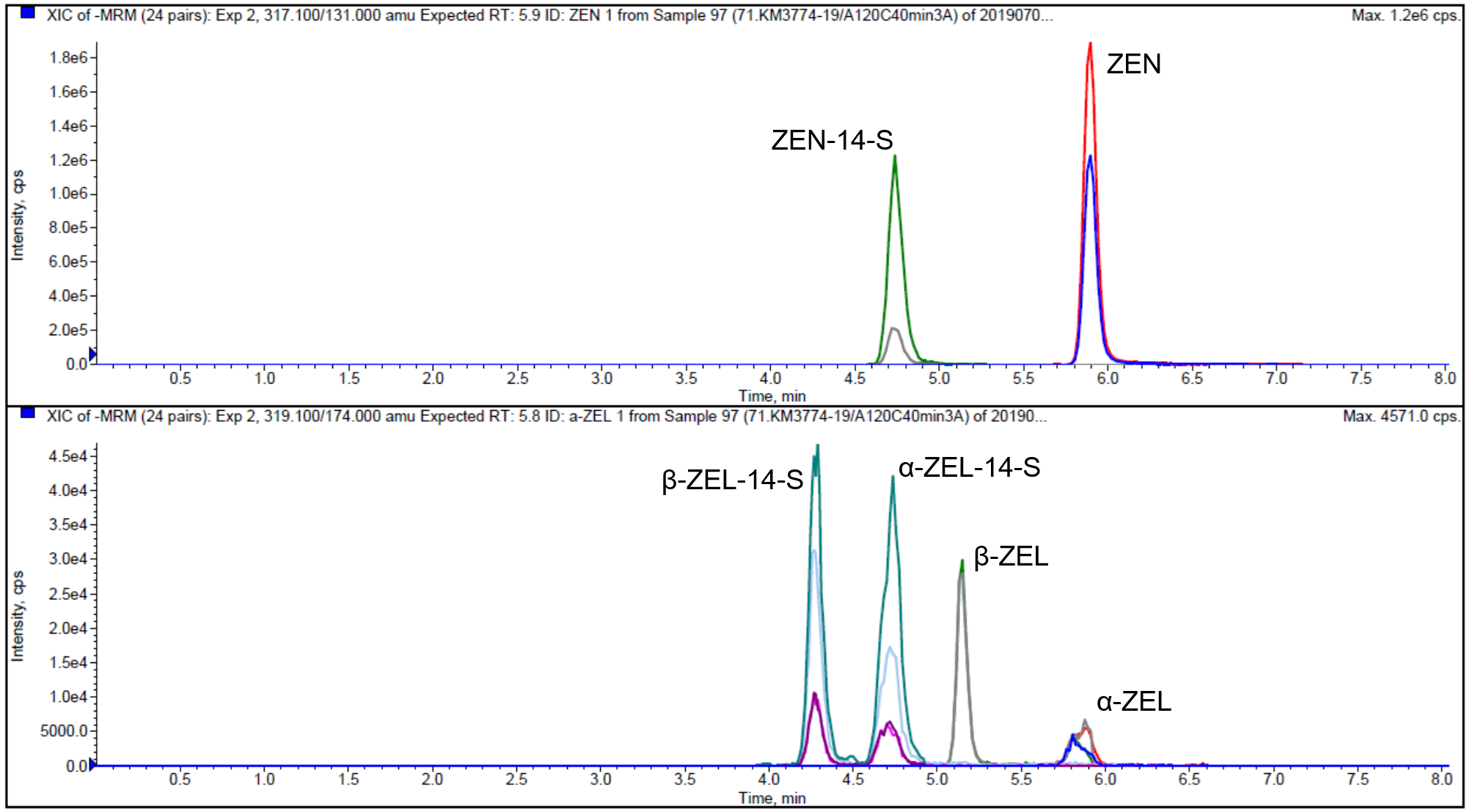


Figure 1: A chromatogram of zearalenone (ZEN), zearalenone-14-sulfate (ZEN-14-S), a-zearalenol (α-ZEL), b-zearalenol (β-ZEL), a-zearalenol-14-sulfate (α-ZEL-14-S) and b-zearalenol-14-sulfate (β-ZEL-14-S). Table 1 of the manuscript includes the data on the ion transitions and other chromatographic conditions.
