# Supplementary material for: Detection and quantification of zearalenone and its modified forms in enzymatically treated oat and wheat flour
Source: J Food Sci Technol. 2023 Feb 15;60(4):1367–75. doi: 10.1007/s13197-023-05683-6 (PMC10020390; doi:10.1007/s13197-023-05683-6)
Supplement: Supplementary file 2 — Supplementary file2 (DOCX 16 KB) [file 13197_2023_5683_MOESM2_ESM.docx]

| **Injections** | **[ZEN-14-S], ng/mL** | **ZEN-14-S quantifier transition** | **α-ZEL-14-S quantifier transition** | **Ratio Q ZEN-14-S / Q α-ZEL-14-S** |
| --- | --- | --- | --- | --- |
|  |  | **Q 397.1-->317.1** | **Q 399.1-->319.1** |  |
|  |  | **Area counts** | **Area counts** | **%** |
| 1st injection | 546.25 | 37584154.9 | 1130214.4 | 3.01 |
|  | 273.13 | 21892781.5 | 677193.0 | 3.09 |
|  | 109.25 | 6744114.5 | 191117.4 | 2.83 |
|  | 54.63 | 3113856.5 | 89708,9 | 2.88 |
| 2nd injection | 546.25 | 40264403.0 | 1118930.6 | 2.78 |
|  | 273.13 | 21233625.2 | 607224.0 | 2.86 |
|  | 109.25 | 6377495.5 | 193574.0 | 3.04 |
|  | 54.63 | 2976873.0 | 89749.6 | 3.01 |
| 1st/2nd injection half volume | 54.63 | 1432884.6 | 42058.8 | 2.94 |
|  | 273.13 | 10308395.1 | 293501.0 | 2.85 |
|  | 54.63 | 1406594.8 | 39085.3 | 2.78 |
|  | 273.13 | 10176209.3 | 306112.6 | 3.01 |
| Average ratio±SD |  |  |  | **2.92±0.11** |

Table 1: Areas of quantifier ions of ZEN-14-S and α-ZEL-14-S and their calculated ratio at different concentrations of the standards and injection volumes

ZEN-14-S – zearalenone-14-sulfate; α-ZEL-14-S – α-zearalenol-sulfate
